# The Relation of the Eye to General Medicine

**Published:** 1912-04

**Authors:** B. Cecil Stockard

**Affiliations:** Atlanta, Ga.


### THE RELATION OF THE EYE TO GENERAL DISEASE.

By B. Cecil Stockard, M. D. Atlanta, Ga.

Several states have, in recent years, passed bills to regulate and license the pracice of so-called optometry, with the avowed intention of preventing incompetent men from traveling over the country fitting glasses upon as many people as will buy them, and then going on to the next town to repeat the performance; and so far it is a good thing, but the public does not distinguish between "optometrist" and "oculist," and are apt to think that opticians are licensed by the state as competent to treat all manner of eye conditions.

It is my purpose in this paper to sketch briefly some of the conditions which are apt to confront an eye-man in which a general knowledge of medicine is essential.

Perhaps the most common are those in which indicanuria is associated with a refractive error. In these cases it is necessary to correct both conditions, that of the eyes, and the digestive disturbances before the headache, eye strain, etc., will disappear.

Indicanuria seems frequently to cause a disturbance of the extraocular muscles, occasionally even a spastic squint, anda also occasionally an impairment of vision due probably to retrobulbar neuritis.

So important is the role played by syphilis in eye troubles that it should be considered as a possible cause in practically every inflammatory condition, especially iritis, ceratitis, chorioiditis, optic atrophy, neuroretinitis and various paralyses.

Rheumatism is also a frequent cause of iritis, as well as some other inflammatory conditions, such as episcleritis, etc.

Gonorrhoeal ophthalmia is, of course, a secondary infection, and scarcely comes under the scope of this paper.

Brights disease is one of the most important to the ophthalmologist because the retina often shows the first signs of the disease. This is also true in the albuminuria of pregnancy, and in these cases recovery usually follows abortion.

Cataracts are frequently found associated with diabetes, as are occasionally retinitis and other serious conditions. Various anaemias give rise to ocular changes, such as neuro-retinitis, authenopia, and the cataracts and other changes of Hookworm diseases as described by Dr. Calhoun.

Consanguinity of parents frequently gives rise to retinitis pigmentosa, though I believe that for some reason this condition is extremely in the negro.

Gynecological conditions are frequently associated with ocular symptoms chiefly myasthenia, and functional disturbances of the retina, the occasionally more serious complications occur. Enlarged prostates and urethral affections are at times associated with similar conditions.

In hysteria, in the eye as elsewhere, almost any subjective symptoms may occur, pains, temporary blindness, and also muscular disturbances, dilated pupils, etc.

The teeth, gastro-intestinal tract, respiratory tract, vascular system, exophthalmic goitre, lymphatic glands, aural disease, adjacent sinuses and diseases of the skin must also be borne in mind in connection with various ocular affections.

Tuberculosis occasionally affects the eyes, and scrofulous children are particularly prone to inflammatory conditions of the cornea, iris, etc.

Meningitis may produce ocular changes of a most distressing type.

Neuro-retinitis may result from a large dose of quinine, a number of cases of neuritis have been reported following the administration of salvarsan. Alcohol and tobacco ambhyopias are fairly common.

Decreased sensitiveness to pressure on the eyeball is said to be sometimes a symptom of beginning tabes.

Irregularity in the size of the pupils has been observed in incipient tuberculosis.

There is another important side to this question, and that is, general or extra ocular symptoms caused by ocular disturbances.

Under this head may be included the referred pains of iritis and glaucoma, but the principal conditions are those caused by refractive errors.

Myopia, or near-sightedness, rarely causes headaches, eyestrain or conjunctivitis. These patients, however are apt to be studious, and rarely enjoy out-of-door sports, because they do not see well enough at a distance, and also the light hurts their eyes as it is not properly focused on the retina.

Hyperopia or farsightedness and also astigmatism, on the contrary, commonly cause conjunctivitis, eyestrain, and headaches. These headaches vary very much in location and degree, usually come on after close work and are sometimes accompanied by nausea, especially where there is astigmatism.

Errors of refraction are the usual cause of strabismus or crossed eyes, the eye usually diverges or turns outward in myopia, and converges or turns inward in hyperopia. It should be remembered that measles, whooping cough, scarlet fever, etc., do not cause strabismus, as parents frequently insist.

I have taken a very general view of this subject with the idea of emphasizing its importance, and showing that ophthalmology should never be considered as a separate profession, but as a more or less important branch of medicine.

Empire Life Building.

